# Optimized cementitious immobilization of simulated cesium and barium radionuclides in borate waste solution by natural zeolite additives

**DOI:** 10.1007/s11356-025-37369-1

**Published:** 2026-01-29

**Authors:** Gabriel Iklaga, Nándor Kaposy, István Tolnai, Zsolt Endre Horváth, Zoltán Kovács, Nedson Theonest Kashaija, Viktória Gável, Csaba Szabó, Margit Fábián, Zsuzsanna Szabó-Krausz, Péter Völgyesi

**Affiliations:** 1 Lithosphere Fluid Research Lab, Institute of Geography and Geology, Eotvos Lorand University, Eotvos Lorand Tudomanyegyetem, Pazmany Peter Setany 1/C, Budapest, 1117 Hungary; 2 HUN-REN Centre for Energy Research: Magyar Kutatasi Halozat Energiatudomanyi Kutatokozpont, Nuclear Security Department, Konkoly Theg M. Ut 29-33, Budapest, 1121 Hungary; 3 Institute of Raw Material Processing and Environmental Technology, Faculty of Earth and Environmental Sciences and Engineering, Miskolc University, Miskolci Egyetem, Miskolc, 3515 Hungary

**Keywords:** Boric acid, Cesium, Barium, Portland cement, Sulfoaluminate cement, Zeolites, Adsorption, Leaching, Immobilization, Radioactive waste, Cementation

## Abstract

This study investigates the immobilization of cesium and barium contaminants in borate waste solutions using clinoptilolite-bearing and mordenite-bearing additives within cement matrices, with a focus on their adsorption capacity. These zeolites were incorporated at varying ratios into blends of Portland cement (PC) and calcium sulfoaluminate cement (CSAC). A batch adsorption experiment was conducted, utilizing borate waste solutions containing trace concentrations of simulated cesium-137 radioisotopes and its metastable decay product barium-137, to evaluate their effectiveness in reducing the leachability of these contaminants after a 28-day solidification period. The experimental setup included a comprehensive analysis involving ASTM standard leaching tests over 11 days, alongside compressive strength testing, scanning electron microscopy (SEM), and X-ray diffraction (XRD) for morphological and chemical assessment at the cement mineral level. Inductively coupled plasma optical emission spectroscopy (ICP-OES) and mass spectroscopy (ICP-MS) analyses were conducted on the leachate from the solidified cement pastes to study the physical and chemical changes of the cement pastes matrices. Results showed that cement paste matrices with untreated clinoptilolite-bearing samples exhibited the highest cesium adsorption capacity in the boric acid liquid waste, whereas KCuHFC-treated zeolite-bearing (i.e., clinoptilolite-bearing and mordenite-bearing) samples showed diminished capacity for cesium adsorption from the boric acid liquid waste. The use of 5% clinoptilolite in the PC/CSAC blend produced optimal physical and chemical stability, highlighting its potential for effective cesium immobilization in waste management.

**Supplementary Information:**

The online version contains supplementary material available at 10.1007/s11356-025-37369-1.

## Introduction

**Fig. 1:**
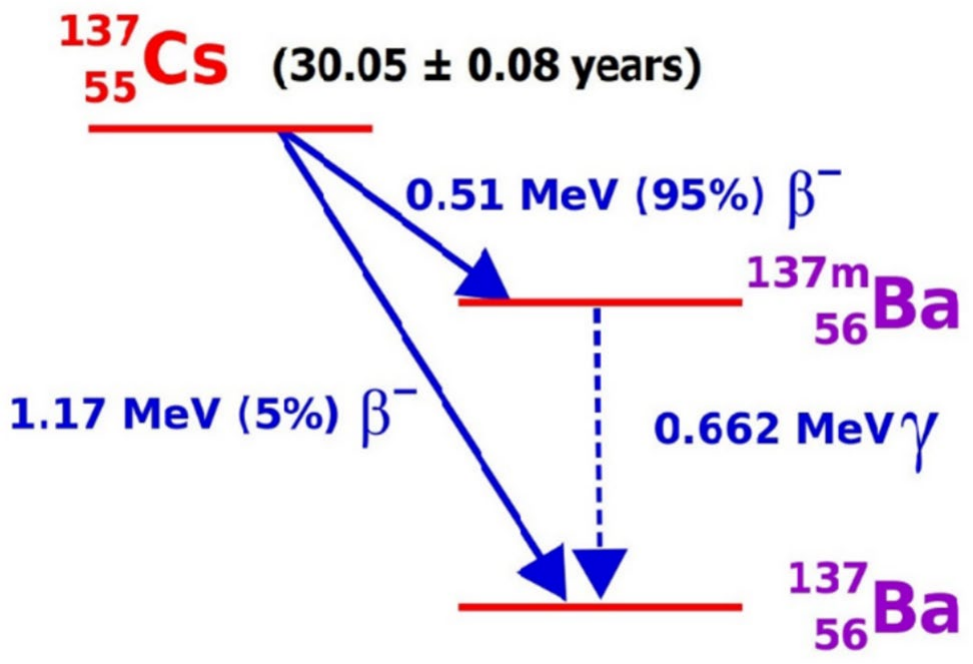
Illustration of ^137^Cs decay scheme

Pressurized water reactors (PWR) use boric acid, which is an intermediate level radioactive waste (ILW) (Sharma et al. 2024; Zatloukalová et al. 2021) since boric acid serves as a potent neutron absorber (i.e., neutron poison) and stable operating temperature facilitator. This high volume waste may contain trace concentrations of radionuclides produced by nuclear fission, such as ^137^Cs (with a ^235^U fission yield of 6.3 mass % (Lehto and Huo 2011) and its decay daughter, ^137m^Ba (also known as ^137m^Cs) (Wang and Zhuang 2019), which has a half-life of approx. 30 years (Delacroix et al. 2002). Another cesium isotope, ^135^Cs, is also produced with a fission yield of 6.5 mass % and 2.3 million years radioactive decay half-life. Its ${\upbeta }^{-}$-decay produces another barium isotope, ^135^Ba. Hence, when the chemical effects of the immobilized Cs radioisotopes are being studied, due to the metastable and, therefore, radioactive ^137m^Ba, it is equally important to concurrently assess the effects of the barium daughter isotopes being produced in the system (Fig. 1).

Improving the storage of borate liquid waste in cement waste forms is topically highly important to the environment because the mentioned radionuclides can enter the biosphere and contaminate food chains through leaching into the natural systems if not effectively immobilized. Radioactive waste is generally categorized into high-level waste (HLW), intermediate-level waste (ILW), and low-level waste (LLW), scaled according to their nuclear activity. The HLW and ILW are restricted to disposal in intermediate depth and deep geological repositories, whereas LLW, e.g., borate waste solution with trace concentrations of fissile radionuclides, is stored in comparatively near-surface repositories (Sharma et al. 2024).

Several studies on the chemical and physical stabilization of borate waste solution in Portland cement (PC) indicated the stability retardation of the resultant waste forms, which might shorten their longevity in repositories (Gorbunova 2015; Rostamiparsa et al. 2023; Sun and Wang 2010). Similarly, previous experimental research studies of calcium sulfoaluminate cement (CSAC) application showed structural weakness in the produced cement paste samples, due to surplus production of sulphates and alumina cement minerals (e.g., ettringite and gypsum), which can be detrimental to the cement structural integrity (Kashaija et al. 2024; J. Li et al. 2021; Pimraksa and Chindaprasirt 2018; Sun et al. 2011, 2014). Our previous study showed that a blend of 80% PC to 20% CSAC is chemically and physically optimal for immobilizing boric acid liquid waste (Iklaga et al. 2025).

Alkali and alkaline earth cations are found in the three-dimensional crystalline frame structures of natural zeolites, which are hydrated alumina-silica based tectosilicate minerals. Zeolites have high micro-porosity of up to 30%, which allows them to both absorb and desorb water. Natural zeolites (like, clinoptilolite and mordenite) have been added to different kinds of cements due to their cation exchange capacity, which depends on the ion species kinds, silica/alumina ratio, and pH of the pore solution of the cement matrices (Abdelrasoul et al. 2017; Abtahi et al. 2018; Bagosi and Csetenyi 1999; Islam et al. 2022; Liguori et al. 2019; Ri et al. 2023; Roshanfekr Rad and Anbia 2021). The adsorption of Cs^+^ from aqueous solutions has been found to be challenging due to its homologous chemical property with other alkali metals like K^+^ and Na^+^. At different pH levels, these monovalent cations do not precipitate with common compounds like halides and sulphate to produce insoluble complexes. Furthermore, with increasing atomic number, they exhibit an increase in crystal ionic radii i.e. Na^+^ (116 pm) < K^+^ (152 pm) < Cs^+^ (181 pm). However, in a completely hydrated state (i.e., hydrated cations with a hydrate shell), they share almost similar sizes, expressed in picometers: Na^+^ (228 pm) > Cs^+^ (219 pm) > K^+^ (212 pm) (Wang & Zhuang 2019). To mitigate these factors, some experimental studies have attempted to improve the ionic exchange capacity of different natural zeolites by impregnating them with metal hexacyanoferrate (Abtahi et al. 2018; Banerjee et al. 2017; Borai et al. 2009; Czapik 2023; Kazemian et al. 2006; Kim et al. 2020; Kivan et al. 2024; Ri et al. 2023; Takahatake et al. 2012; Zheng et al. 2024). However, a close observation of related literature shows that not a significant attention has been given to the adsorption characteristics of natural zeolite additives in cementitious blends used to immobilize boric acid solutions, containing trace concentrations of Cs and Ba ions.

This study focused on assessing the adsorption capacity of mordenite and clinoptilolite collected in rhyolitic tuff samples and their geochemical effects, as additives, in a blend of PC and CSAC used for enhancing the immobilized borate waste solution that contains trace concentrations of cesium (simulating ^137^Cs) and its daughter barium (simulating ^137m^Ba) in cement waste.

## Materials and methods

### Experimental flowchart of the research

**Fig. 2:**
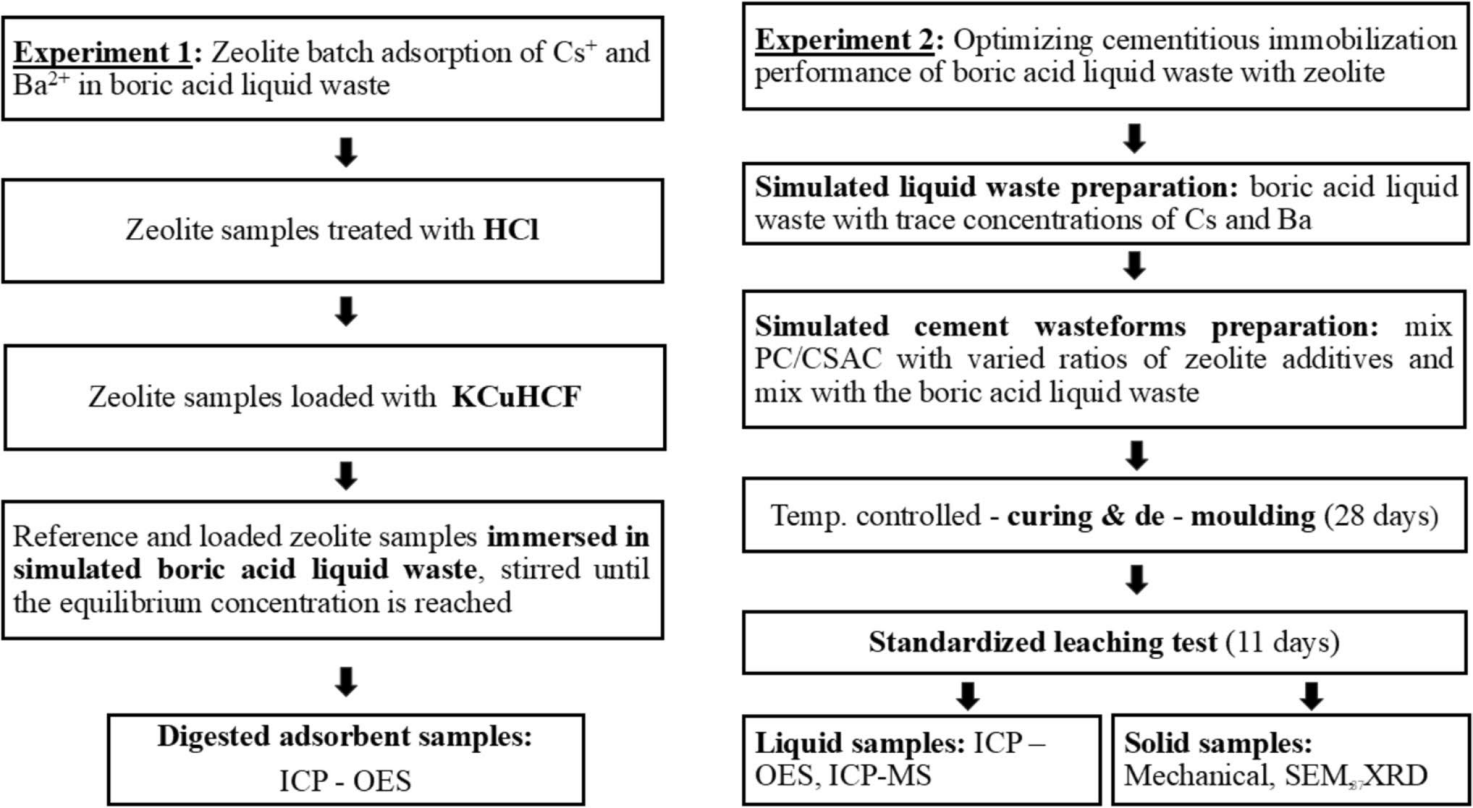
Experimental procedure chart of the study. Where: KCuHCF denotes potassium copper (II) hexacyanoferrate; HCl, hydrochloric acid; PC, Portland cement; CSAC, sulfoaluminate cement; zeolite samples: clinoptilolite-bearing and mordenite-bearing additives; ICP-OES, inductively coupled plasma-optical emission spectrometry; ICP-MS, inductively coupled plasma mass spectrometry; SEM, scanning electron microscope, and XRD, X-ray diffraction

The flowchart of this research was segmented in two phases, as depicted in Fig. 2: column (1) represents the batch adsorption test for evaluating barium and cesium adsorption in borate waste solution using clinoptilolite-bearing and mordenite-bearing additives; column (2) denotes the enhancement of borate waste solution in a blend of PC and CSAC with clinoptilolite-bearing and mordenite-bearing additives to improve the encapsulation of cesium in the cement matrices.

### Zeolite-bearing samples collection

Zeolite-bearing rhyolite tuff samples were collected in Tokaj Mountain situated in northeastern Hungary, with volcanological formation dated between 11 and 13 million years ago due to felsic volcanism at the boundaries between the great Hungarian plains and Carpathians. Tokaj Mountains has been known for retaining zeolite-rich rhyolite tuff (Zajzon et al. 2021). In this study, zeolite-bearing (rhyolite tuff) samples were collected from two quarries located in the Tokaj region. The Rátka quarry, operated by JOSAB Hungary Ltd. (geographical coordinates: 48.21186, 21.23374), where the zeolite is clinoptilolite (ZC), and the Bodrogkeresztúr quarry, operated by COLAS Hungary Ltd. (geographical coordinates: 48.17386, 21.33974), where the zeolite is mordenite (e.g., Zajzon et al. 2021) (ZM).

### Preparation of borate waste solution

To prepare the borate solution, 40,000 mg of boric acid (produced by NUKEM, with CAS Number: 1333-73−9, composition 80% ^11^B and 20% ^10^B) was used; then, 400 mg of cesium nitrate (produced by Thermo Scientific, with CAS Number: 7789-18−6, composed of ≥ 99.99% (metals basis) CsNO₃) was used to simulate the trace presence of cesium in the waste solution, and 400 mg of barium nitrate (produced by AVANTOR, with CAS Number: 10022-31−8, composed of ≥ 99.99% Ba(NO₃)₂) was used to simulate the trace presence of barium in the waste solution. Crystalized powders of the stated reagents were mixed with 1 dm^3^ of demineralised water (conductivity = 1.1 S/cm^−1^, pH = 7.5 at 23 °C) to replicate the boric acid waste conditions from PWRs (i.e., with simulated concentrations ⁓ 40,000 ppm boron, ⁓ 400 ppm cesium, and ⁓ 400 ppm barium). Additionally, sodium hydroxide (produced by AnalaR NORMAPUR, VWR Bdh, with CAS Number: 1310-73−2, composed of 99.2% purity NaOH) was employed to neutralize boric acid, producing a borate solution with a 1.25 mmol unit of H_3_BO_3_/NaOH ratio for enhancing the solubility of the resulting waste solution and mitigating cement hydration retardation, as the resultant increased pH of the borate solution optimizes the curing of the cement paste (Iklaga et al. 2025; Rostamiparsa, 2023).

### Simulated cement wasteforms

#### Composition of cement used in the experiments

In this study, two types of cements were used for making the waste samples. These include calcium sulfoaluminate cement (CSAC) (grade R – 14734 ALI CEM GREEN) and Portland cement (PC) (grade R – 15796 CEM I 52.5 N), which were both acquired at CEMKUT company Hungary. The chemical and mineral characterization of both types of cements was identical and consistent with our prior research conducted under the same testing standard (EN 196–2–2013) (Iklaga et al. 2025).

#### Preparation of cement paste sample blend with zeolite additives

The cement paste ratio of PC 80 mass % to CSAC 20 mass % was taken as the optimal cement blend for the immobilization of boric acid liquid waste based on our previous research results and used as the benchmark for the PC/CSAC blend before the internal addition of the natural zeolite-bearing (i.e., clinoptilolite ZC and mordenite ZM) samples in ratios 0, 5, 10, and 15 mass %. The enhanced cement blend for encapsulating borate waste solution, based on prior research (Iklaga et al. 2025), was determined to be a cement blend ratio of CSAC 20 mass % to PC 80 mass %. This blend served as the standard CSAC to PC mix prior to the incorporation of clinoptilolite-bearing and mordenite-bearing additives in ratios of 0 mass %, 5 mass %, 10 mass %, and 15 mass %. The procedure for mixing the cement, zeolites, and borate waste solution (the solution-to-solid ratio used was 0.4), as well as curing of the resultant cement waste forms in vessels to form moulds before analyses, followed the same laboratory protocols and apparatus used in our previous experimental research (Iklaga et al. 2025).

### Standardized leaching experiment

In compliance with the ASTM (American Society for Testing and Materials) C1308-08 (ASTM Internationalinternational 2017), standardised leaching tests were carried out. Following their immersion in 500 cm^3^ of DM water (leachant), the resultant 28 days cured cement moulds shaped as cylinders, having a total surface area of 50 cm^2^ were precisely changed at 2, 5, 17, and 24 h, as well as every day for the following ten days. Furthermore, the pH values of all the resultant leachates were already published (Iklaga et al. 2025; Rostamiparsa et al. 2023). After the leaching test, the pH of the leachates was measured, and along with the cement paste, samples were stored in airtight vessels before further analytical measurements.

### The batch adsorption experiment

The standard batch adsorption procedure was conducted to evaluate the adsorption potential of Cs^+^ and Ba^2+^ (Fig. 1) in borate waste solution using clinoptilolite-bearing and mordenite-bearing samples. Additionally, the clinoptilolite-bearing and mordenite-bearing samples underwent treatment as they were loaded with potassium copper (II) hexacyanoferrate (KCuHFC, CuSO_4_ + K_4_Fe(CN)_4_) to evaluate the potential of enhancing their selectivity for Cs^+^ and Ba^2+^ (Banerjee et al. 2017; Kazemian et al. 2006; Ri et al. 2023; Voronina et al. 2017; Wang and Zhuang 2019).

#### Materials for the batch adsorption experiment

The clinoptilolite-bearing and mordenite-bearing samples were ground to a grainsize of 200 μm (Ri et al. 2023) before being treated for 24 h in 0.5 M hydrochloric acid solution, sieved, and desiccated to remove carbonate phases from the zeolite samples. Furthermore, the zeolites were submerged for 3 h and stirred at a speed of 530 rotations per minute at a temperature of 80 °C in 0.5 M CuSO_4_ (produced by AnalaR NORMAPUR VWR chemicals, with CAS Number: 7758-98−7, copper (II) sulphate) solution to promote sorption of copper in the pores of the clinoptilolite and mordenite before being sieved and desiccated. After this, the clinoptilolite-bearing and mordenite-bearing samples were loaded for 24 h at 20 °C at a regulated mixing speed of 530 rpm in 0.5 M K_4_Fe(CN)_6_ (produced by GPR RECTAPUR VWR chemicals, with CAS Number: 14459-95−1, Potassium hexacyanoferrate (II) trihydrate) solution before being sieved and desiccated. This resulted in the KCuHFC-loaded clinoptilolite-bearing and mordenite-bearing samples.

#### Protocol for the batch adsorption experiment

Both untreated and KCuHFC-loaded clinoptilolite-bearing and mordenite-bearing samples were submerged for 3 h and mixed at 150 rpm in the borate waste solution to attain equilibrium concentration. The solid and liquid phases were filter separated and stored for further analyses. Before ICP-OES measurement, 0.25 g of each of the resultant solid phase adsorbates was digested in 9 mL HCl (35–37%), 3 mL HNO_3_ (70%), and 2 mL HF (49%) using PerkinElmer MPS 320 system High Pressure 100 mL vessels.

### Methods for characterization and analyses of the zeolites, cementitious samples, and leachates

The zeolite-bearing rhyolite tuff samples (i.e., clinoptilolite-bearing and mordenite-bearing samples) collected onsite were analyzed to verify their mineralogical compositions, morphological, and elemental characteristics.

#### Mechanical test

A compressive strength experiment following the European standard protocol EN 196-1:2016 E (European Committee for Standardization, 2016) was conducted to examine variations in the mechanical strength of cement waste forms with varying ratios of clinoptilolite-bearing and mordenite-bearing additives. The compressive strength was measured using a calibrated pressing machine from Toni Technik Baustoffprüfsysteme GmbH provided by CEMKUT Research Laboratory, Budapest. The cement samples were measured following exact protocols mentioned in our previous publication (Iklaga et al. 2025).

#### Scanning electron microscope (SEM)

A Hitachi TM 4000 plus model SEM was utilised to acquire morphological data of the solidified cement samples. Because cement samples are porous, SEM images were acquired in backscattered electron detector (SEM-BSE) mode that operates at 10 kV in low vacuum. For the analyses, the surfaces of the sample cross sections were polished. The analyses were performed at the SEM laboratory at Eötvös Loránd University, Budapest, Hungary. The cement samples were cut, and the surface polished before the SEM analysis. An image processing software (ImageJ, version 1.38e/Java 1.5.0_09) was used to measure the unreacted anhydrous mineral phases on the SEM-BSE images of the cement samples (Gaël et al. 2016) using a threshold brightness histogram algorithm (Iklaga et al. 2025). To get the uncertainty values, each sample was measured three times, with an average uncertainty percentage of 10%. The sample preparation for the SEM was done at the Lithosphere Fluid Research Laboratory at Eötvös Loránd University, Budapest, Hungary.

#### X-ray diffraction (XRD)

The pulverized cement samples were mineralogically analyzed using X-ray diffractometry (Bruker AXS D8 Discover with Cu Kα radiation source, *λ* = 0.154 nm), which was equipped with a Göbel mirror and scintillation detector (Bruker GmbH, Karlsruhe, Germany). The equipment operated at 40 kV and 40 mA. Diffraction patterns were obtained within a 2θ range of 5° to 65° using a step size of 0.02°. The Diffrac.Eva software was utilized to analyze the measured XRD patterns and identify the characteristic crystalline mineral phases at the Institute of Technical Physics, HUN-REN Centre for Energy Research, Budapest, Hungary. The powdered cement paste waste forms after the standardized leaching test were used for this analysis.

#### Inductively coupled plasma-optical emission spectrometry (ICP-OES)

PerkinElmer Avio 200 ICP-OES with generator power of 1500 W, plasma gas flow rate: 12 l/min, sample flow rate: 1 mL/min was used. For the digested samples from the batch adsorption experiment and leachate samples from the standardized leaching experiment, the cesium line was measured in axial view, every other element was measured in radial view, with Yttrium (Y) used as the internal standard. The MSZ EN ISO 11885:2009 standard was applied as the measurement procedure. The measurement uncertainty was ± 15%. This analytical measurement was conducted at the Institute of Technical Physics, HUN-REN Centre for Energy Research, Budapest, Hungary.

#### Inductively coupled plasma mass spectrometry (ICP-MS)

PerkinElmer NexION 1000 multi-quadrupole ICP-MS using He kinetic energy discrimination KED interference correction via its Universal Cell Technology component UTC and Rhodium-103 (^103^Rh) as internal standard was also used as a further verification analytical method for measuring the elements of interest in the leachate samples from the standardized leaching experiment. The MSZ EN ISO 17294-2:2017 standard was applied as the measurement procedure. The measurement uncertainty was ± 10%. This analytical measurement was conducted at the ICP-MS laboratory at the Geological Survey of the Supervisory Authority for Regulatory Affairs, Budapest, Hungary.

### Calculation methods

Following the standardized leaching test experiment and inductively coupled plasma, optical emission spectroscopy (ICP-OES) analyses, the incremental fraction leached (IFL), and cumulative fraction leached (CFL) formulae shown in Eq. (1) and Eq. (2), respectively, as in our previous studies (Iklaga et al. 2025; Rostamiparsa et al. 2023) were used to calculate the fraction of elemental cesium leachability diffused out of the cement paste samples (ASTM Internationalinternational 2017).

#### Incremental fraction leached (IFL)

This method calculates the unitless incremental fraction leached (IFL_n_) of cesium during the nth test interval as shown in Eq. 1:

1 $$IFL_n=a_n^{Cs}/A_0^{Cs}$$

where ${{\boldsymbol{a}}}_{{\boldsymbol{n}}}^{{\boldsymbol{C}}{\boldsymbol{s}}}$ (mg/L) is the amount of cesium determined in the *n*^*th*^ test interval leachate, ${{\boldsymbol{A}}}_{0}^{{\boldsymbol{C}}{\boldsymbol{s}}}$ (mg/L) is the amount of cesium present in the solidified cement material before the leaching test.

#### Cumulative fraction leached (CFL)

Equation (2) calculates the cumulative fraction of elemental cesium leachability (CFL_j_) up until the *j*th interval:

2 $$CFL_j=\sum\nolimits_{n=1}^ja_n^{Cs}/A_0^{Cs}=\sum\nolimits_{n=1}^jIFL_n$$

Graphically comparing the CFL data with the progressing time during the leaching experiment of cement samples, therefore, indicates the characteristic elemental leachability from each cement matrix. 

## Results

### Characterization of the clinoptilolite-bearing (ZC) and mordenite-bearing (ZM) samples

**Fig. 3:**
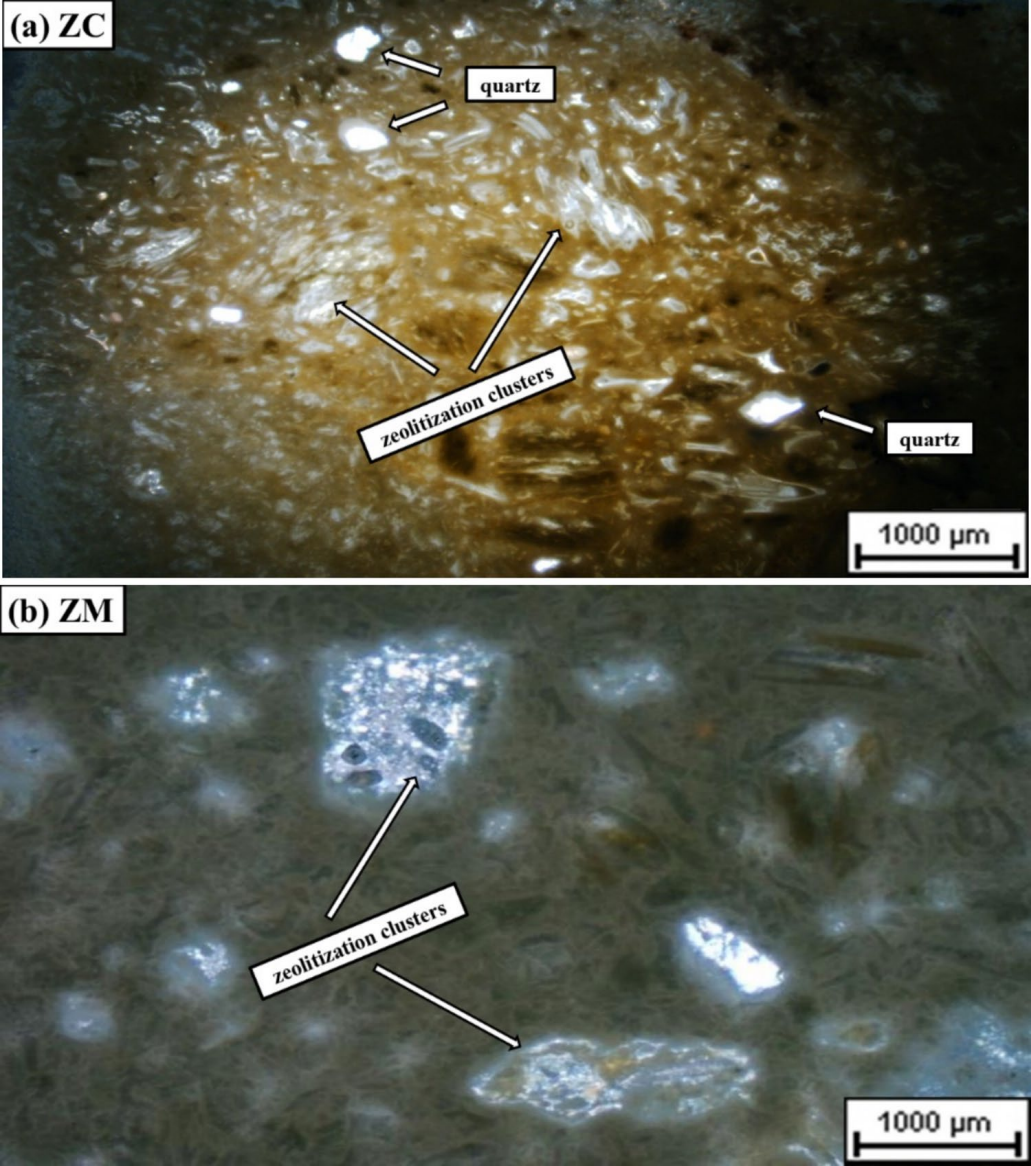
Photomicrographs showing clusters of zeolites (**a**) in clinoptilolite-bearing and (**b**) mordenite-bearing samples, crossed nicols, 10 × magnification

Based on the petrographic observations by the polarized light microscope on samples ZC and ZM, plagioclase and bony-shaped volcanic glass fragments can be identified in both samples. The characteristic volcanic glass fragments partially went through the zeolitization process, showing clusters of zeolites (Fig. 3a, b). Additionally, quartz and feldspar were also observed in both samples.

**Fig. 4:**
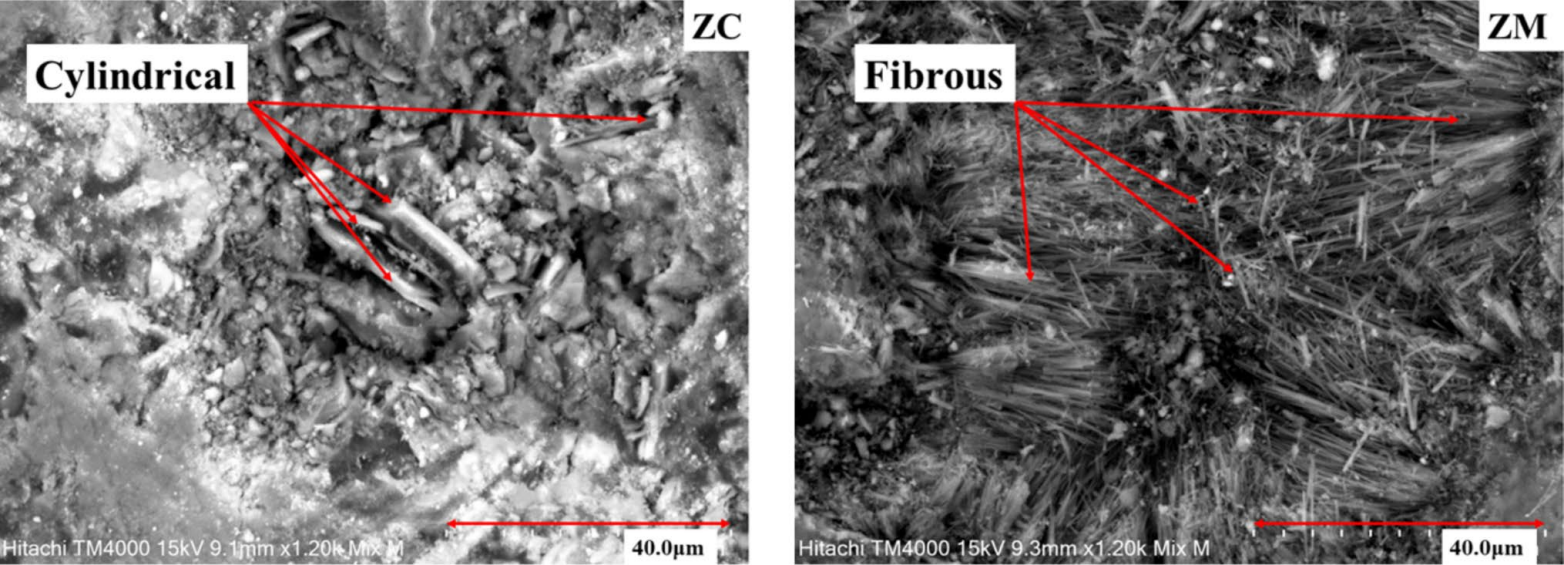
Morphological characterization images, using scanning electron microscopy secondary electron (SEM-SE) show flaky and cylinder-shaped grains in clinoptilolite-bearing sample (ZC) and fibrous patches in the mordenite-bearing sample (ZM) sample

Morphology characterization of zeolites, using scanning electron microscopy secondary electron (SEM-SE) images, the clinoptilolite-bearing sample (ZC) is dominated by flaky and cylinder-shaped grains, whereas mordenite-bearing sample (ZM) contains a significant amount of fibrous grains (Fig. 4a, b).

**Fig. 5:**
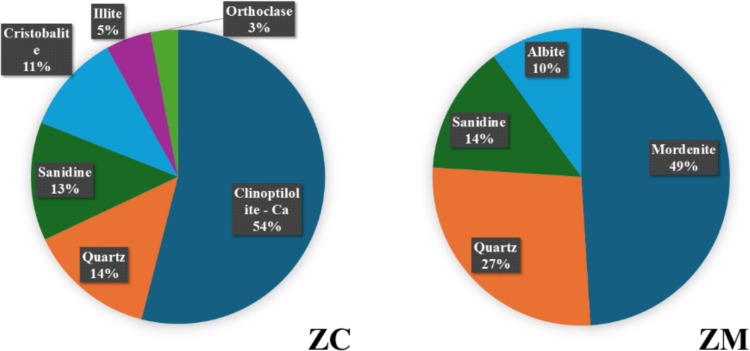
X-ray diffraction characterization of clinoptilolite-bearing ZC and mordenite-bearing ZM samples showing the detected minerals

The X-ray diffraction characterization of sample ZC consists of 54 weight percent (wt. %) clinoptilolite Ca_3–6_(Si_30_Al_6_)O_72_.20H_2_O, 16 wt. % K-feldspar, 14 wt. % quartz, 11 wt. % cristobalite, and 5 wt. % illite per bulk sample of ZC. Whereas sample ZM contains the presence of 14 wt. % K-feldspar, 27 wt. % quartz, 10 wt. % plagioclase, with 49 wt. % mordenite (Na_2_, Ca, K_2_)_4_(Al_8_Si_40_)O_96_.28H_2_O per bulk sample of ZM (Fig. 5).

Inductively coupled plasma mass spectrometry (ICP-MS) measurement of both zeolite-bearing samples shows a significant concentration of Al and Fe, and trace amounts of Ba (59.5 ± 1.7 mg/kg), B (2.85 ± 0.32 mg/kg), and Cs (0.631 ± 0.052 mg/kg) in ZC and of Ba (15.2 ± 0.7 mg/kg), B (3.18 ± 0.53 mg/kg), and Cs (0.465 ± 0.064 mg/kg) in ZM sample (Table 1).

**Table 1:** ICP-MS analyses of clinoptilolite-bearing (ZC) and mordenite-bearing (ZM) samples showing elemental concentrations in mg/kg. Where: ZC = clinoptilolite-bearing sample; ZM = mordenite-bearing sample; < LOQ = below the limit of quantification

Element (mg/kg)	ZC	ZM
Al	25,292 ± 1624	9615 ± 1577
Fe	5431 ± 669	5404 ± 902
Sr	356 ± 11.0	38.8 ± 6.5
Ba	59.5 ± 1.7	15.2 ± 0.7
Cs	0.631 ± 0.052	0.465 ± 0.064
Mn	37.4 ± 3.6	77.6 ± 6.3
Zn	28.1 ± 4.0	36.3 ± 3.6
Pb	5.44 ± 0.20	13.8 ± 0.90
B	2.85 ± 0.32	3.18 ± 0.53
Cu	2.28 ± 0.11	16.30 ± 0.60
Cr	1.74 ± 0.28	4.37 ± 0.39
Ni	1.64 ± 0.05	2.30 ± 0.14
V	1.60 ± 0.34	1.95 ± 0.20
Co	0.563 ± 0.054	0.411 ± 0.041
Mo	< LOQ	< LOQ
Cd	< LOQ	< LOQ
As	< LOQ	< LOQ

### Results of batch adsorption experiment

The ICP-OES elemental analyses indicated a quantitative variation in the concentration of Cs^+^ and Ba^2+^ in the borate waste solution adsorbed by the clinoptilolite-bearing and mordenite-bearing samples during the batch adsorption experiment are shown in Table 2.

**Table 2:**
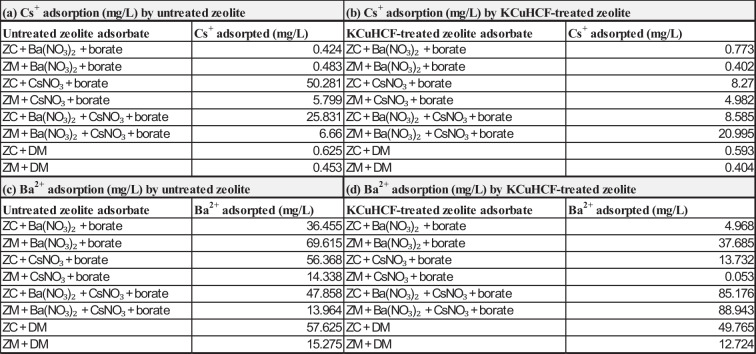
Percentages of adsorption concentration (mg/L) by untreated and KCuHCF-treated clinoptilolite-bearing (ZC) and mordenite-bearing (ZM) samples in simulated borate waste solutions. KCuHCF denotes potassium copper (II) hexacyanoferrate

### SEM results of the cement samples

**Fig. 6:**
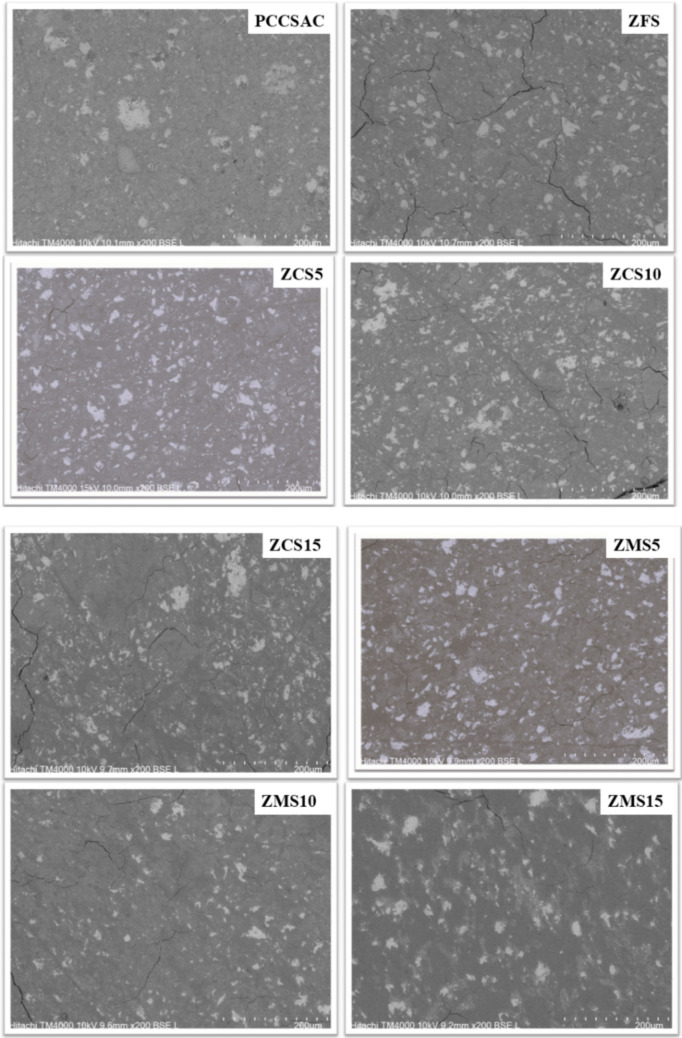
SEM-BSE images of cement samples post leaching test, illustrating alterations in surface morphology of the polished cross-sections of cement paste samples. Where PCCSAC: cement blend + DM water; ZFS: cement blend + boric acid solution; ZCS5: 5% clinoptilolite in cement blend; ZCS10: 10% clinoptilolite in cement blend; ZCS15: 15% clinoptilolite in cement blend; ZMS5: 5% mordenite in cement blend; ZMS10: 10% mordenite in cement blend; and ZMS15: 15% mordenite in cement blend

**Fig. 7:**
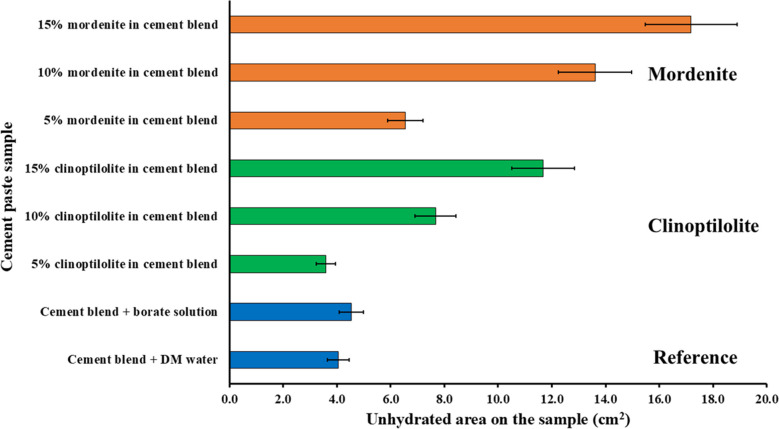
Results of analysis of SEM-BSE images with ImageJ software. Area analyses identified the anhydrous mineral phases, denoted by bright surface hues in Fig. 6

The microstructural variations of the reference cement samples without the zeolite-bearing additives and those cement samples with clinoptilolite-bearing or mordenite-bearing additives were studied by SEM-BSE). This semiquantitative method was utilized to study the surface morphology variations associated with different quantitative ratios of the zeolite-bearing samples in the cement matrices. The anhydrous minerals (appearing bright grains) on the surface of the cement samples (shown in Fig. 6) were used to estimate and quantify the total surface area of unhydrated cement minerals in the SEM-BSE image of the cement samples (shown in Fig. 7 and Table 3) (Gaël et al. 2016). This served as a metric to assess the impact of clinoptilolite or mordenite, and borate waste solution on the hydration rates of each cement sample. The cement sample containing a 5% clinoptilolite exhibited the lowest area of unreacted cement minerals. The results were based on three measurements per sample; the measurement’s average uncertainty was 10%.

**Table 3:**
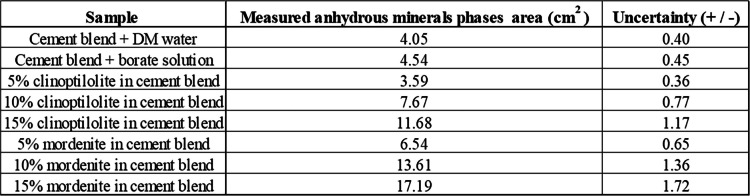
Quantification results of the anhydrous mineral phases (denoted by bright surfaces on the cement paste samples in Fig. 6) area of the SEM-BSE images of the cement paste samples using ImageJ software

### Mechanical test experiment results

**Fig. 8:**
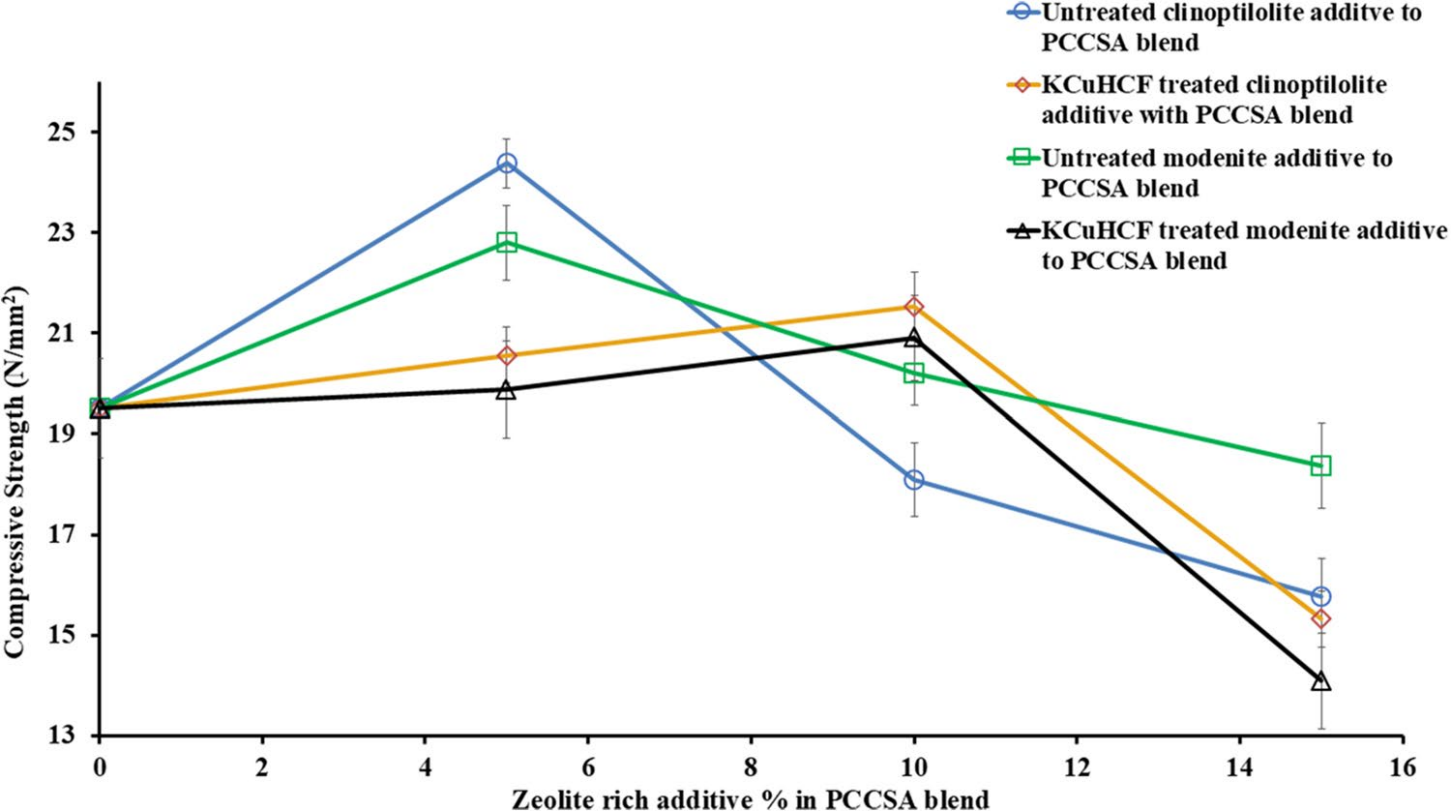
Compressive strength vs zeolite-rich additive % in PCCSAC blend, i.e., 80% Portland cement + 20% sulfoaluminate cement; KCuHCF, potassium copper (II) hexacyanoferrate

The compressive strength test result (shown in Fig. 8 and Table 4) of the cement-paste samples with untreated and KCuHCF-treated zeolite additives (i.e., clinoptilolite and mordenite) showed that the samples with untreated zeolite additives have the highest compressive strength values of 24 N/mm^2^ at 5% of untreated clinoptilolite-bearing additive in the cement matrices. The results showed a decrease in the compressive strength after the 5% zeolite additive mark. To obtain the uncertainty values, four replicas of each cement paste specimen were prepared.

**Table 4:**
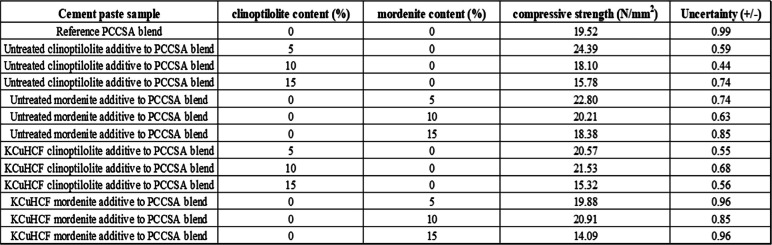
Compressive strength results of the cement paste blend (PCCSA) with increasing quantitative ratio of zeolite-bearing additives (i.e., clinoptilolite-bearing and mordenite-bearing additives). Where PCCSA: 80% Portland cement + 20% sulfoaluminate cement; KCuHCF: potassium copper (II) hexacyanoferrate

### XRD results

**Fig. 9:**
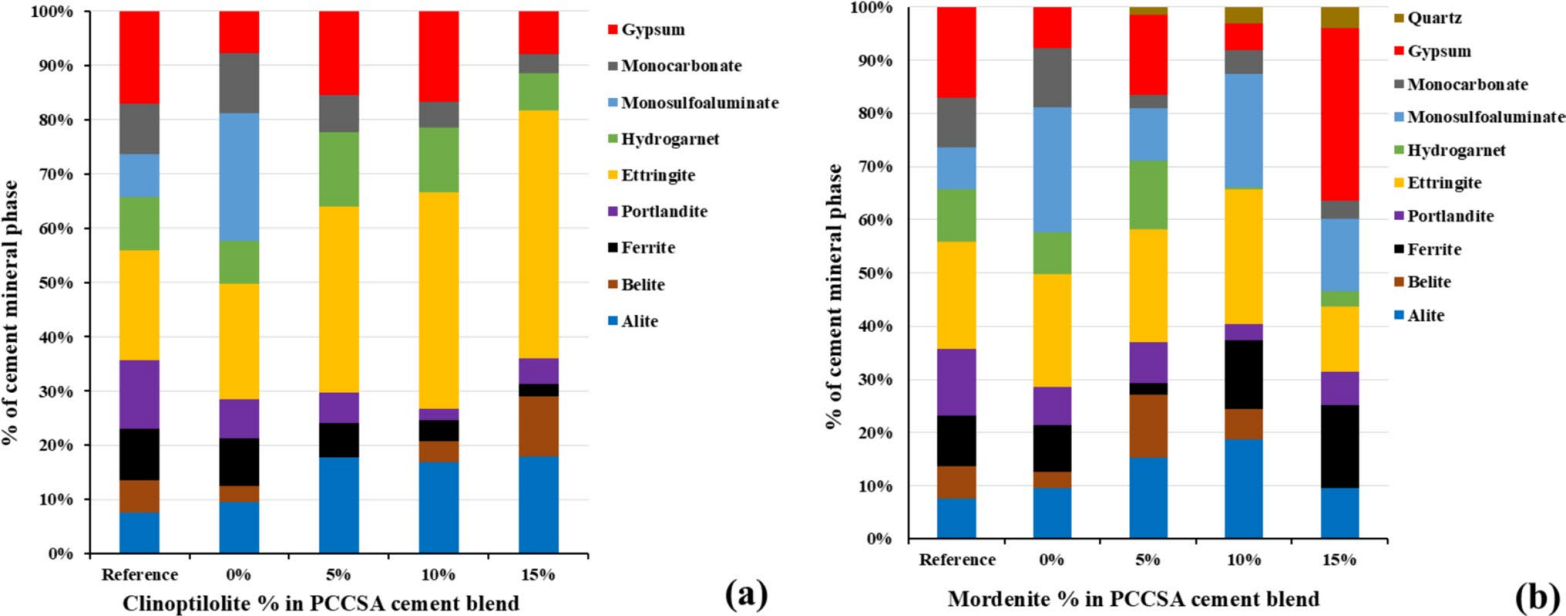
X-ray diffraction results** (**XRD) of the cement paste samples showing percentile (%) changes of cement mineral phases with increasing zeolite-bearing additive quantity (i.e., **a** clinoptilolite; **b** mordenite) in the cement paste blend after 28 days of curing the studied samples. Where PCCSAC: 80% Portland cement + 20% sulfoaluminate cement; Reference: cement blend + DM water

The X-ray diffraction (XRD) results of the powdered cement paste samples indicated that the type and quantity of zeolite-bearing additive (i.e., clinoptilolite-bearing ZC and mordenite-bearing ZM) added to the cement matrices resulted in significant mineralogical changes, reflected in the production and percentage variations of the cement mineral phases (Table 5 and Fig. 9a and b). The mineralogical composition of the cement-paste samples with clinoptilolite-bearing and mordenite-bearing additives can be described as the mixture of the following mineral phases with different proportions: alite Ca_3_SiO_5_, belite Ca_2_SiO_4_, ferrite Ca_2_(Al, Fe)_2_O_5_, portlandite Ca(OH)_2_, ettringite Ca_6_Al_2_(SO_4_)_3_(OH)_12_.26H_2_O, hydrogarnet Ca7(SiO4)3(OH)2, monosulfoaluminate Ca_4_Al_2_(SO_4_)(OH)_12_∙6H_2_O, monocarbonate Ca_4_Al_2_(CO_3_)(OH)_12_.5H_2_O, gypsum CaSO4.2H2O, quartz SiO_2_.

**Table 5:**
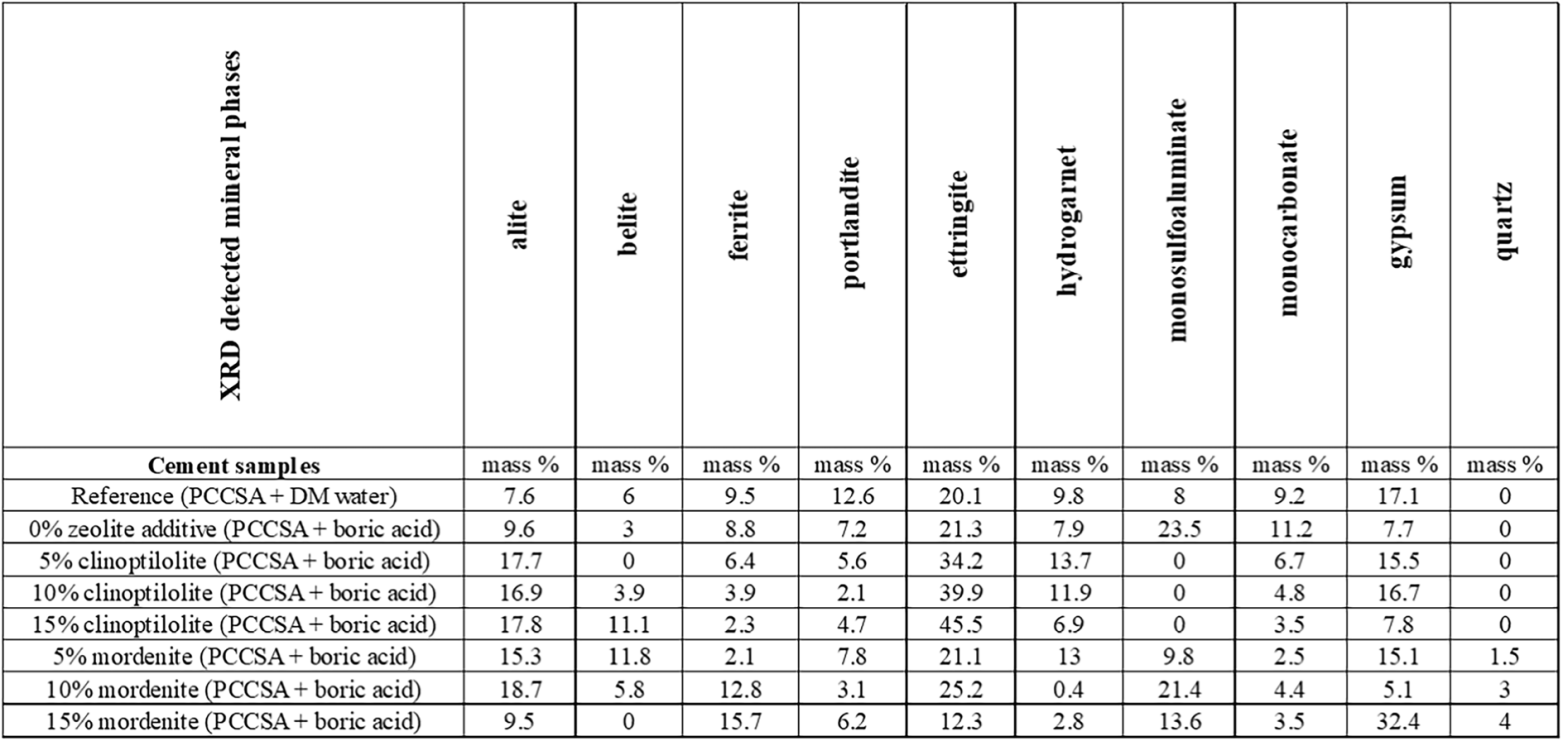
X-ray diffraction results (XRD) of the cement paste samples showing mass % mineral phases detected with increasing zeolite-bearing (i.e., clinoptilolite and mordenite) additives. Where PCCSAC denotes 80% Portland cement + 20% sulfoaluminate cement; reference denotes cement blend + demineralized (DM) water

### The pH measurement results on leachate samples

**Fig. 10:**
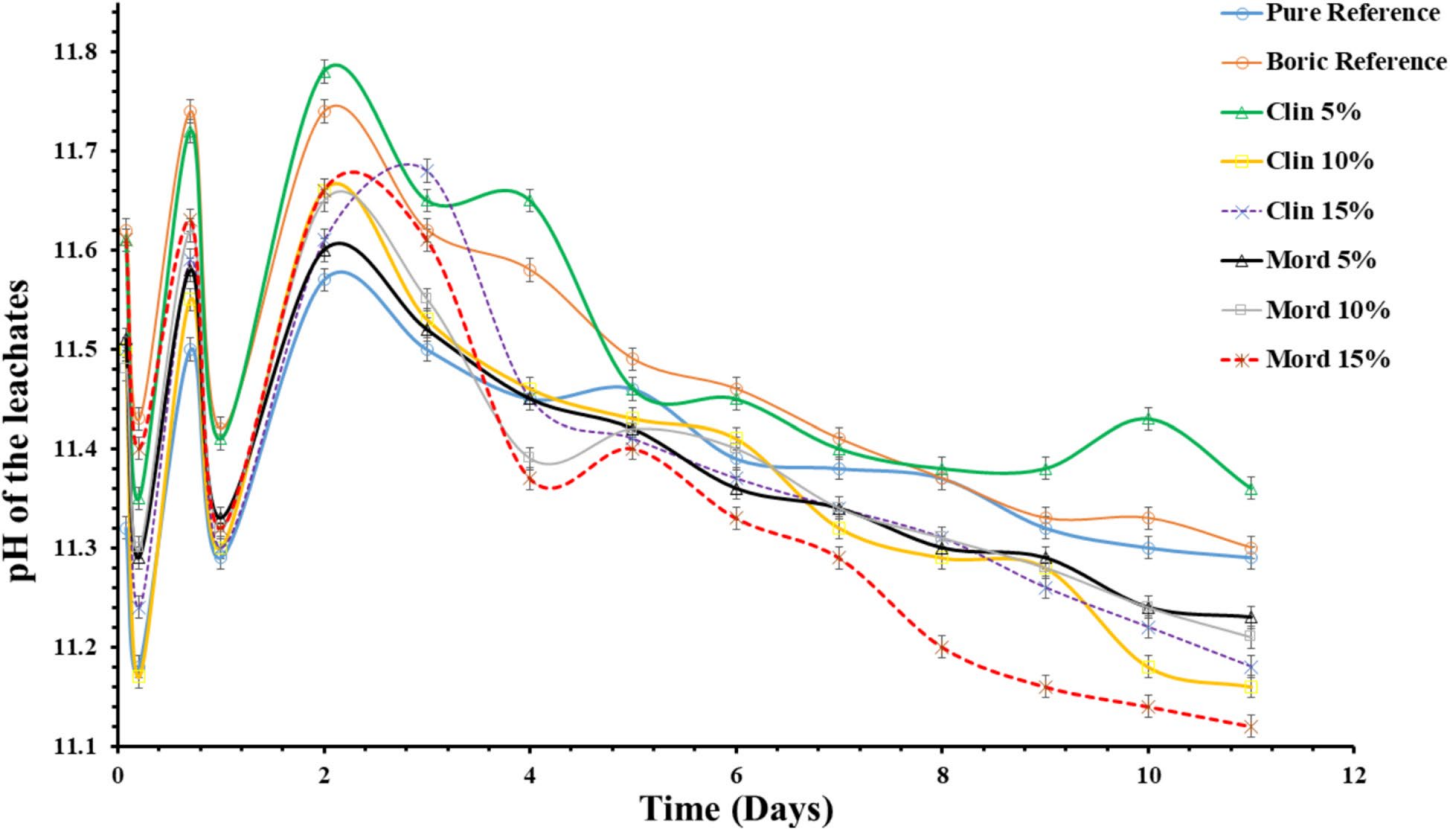
pH values of leachates from cement samples obtained during the leaching test. Pure reference: cement paste blend + demineralized (DM) water; boric reference: cement paste blend + simulated boric acid solution; Clin, clinoptilolite-bearing zeolite additive; and Mord, mordenite-bearing zeolite additive

**Fig. 11:**
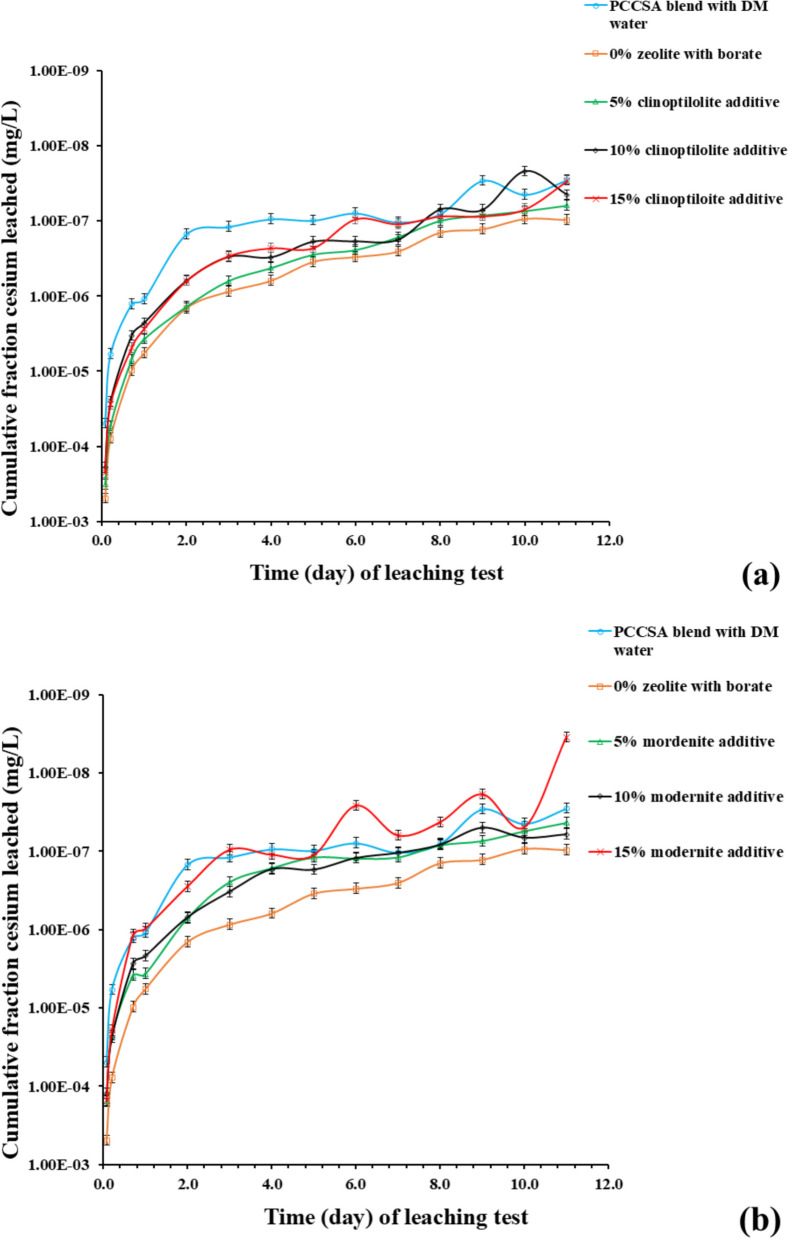

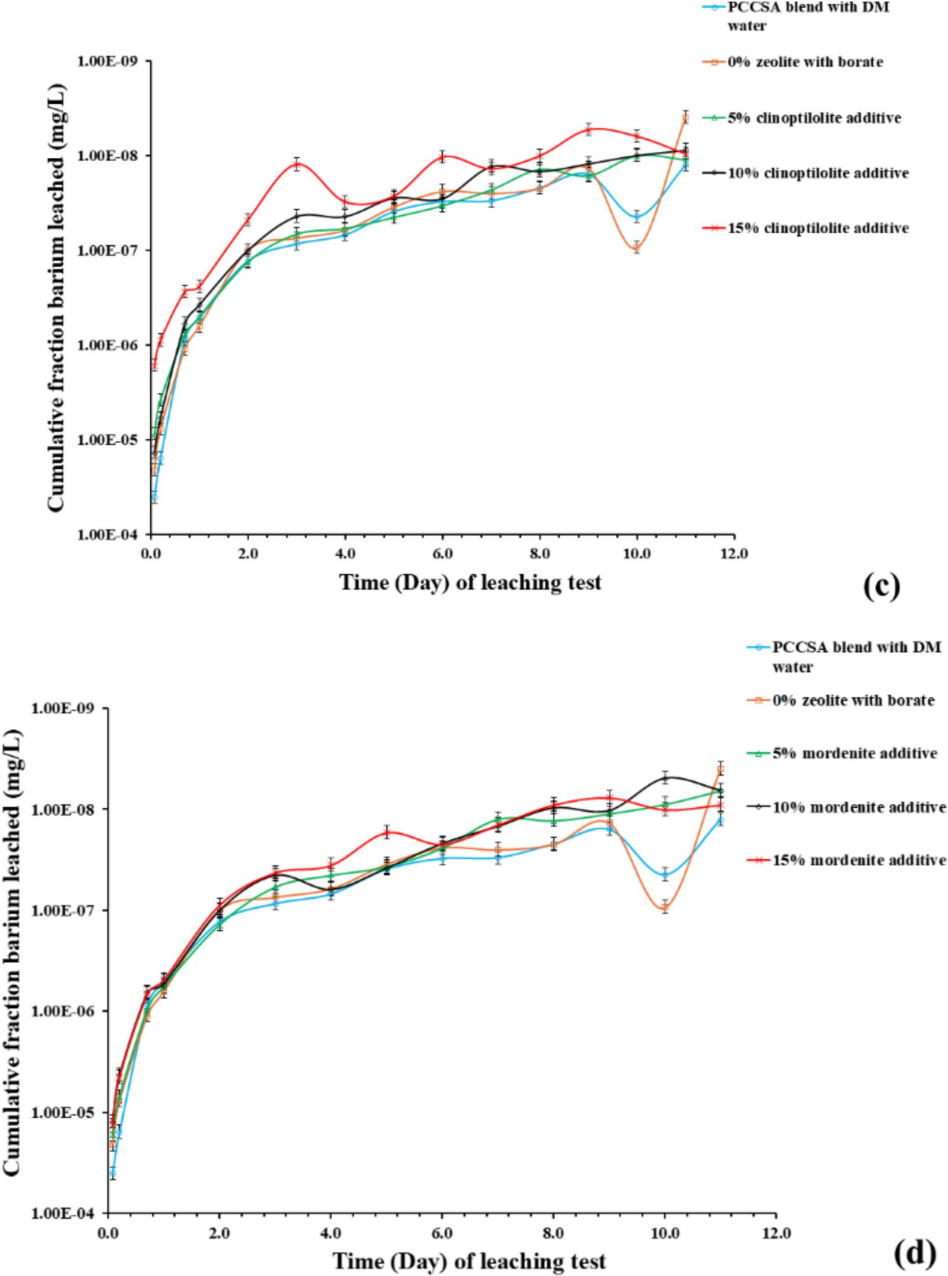
ICP-OES results of elemental cesium leachability from varying ratios of zeolite-bearing additives in the cement blend, **a** PCCSAC blend + clinoptilolite, **b** PCCSAC blend + mordenite; and elemental barium leachability from varying ratios of (**c**) PCCSAC blend + clinoptilolite, **d** PCCSAC blend + mordenite; against leaching experiment time. The ICP-OES measurements for barium and cesium exhibited an average uncertainty of 10%. Where PCCSAC: 80% Portland cement + 20% sulfoaluminate cement; and DM water: demineralized water

The pH measurements of each leachate sample (Table 6) taken during the leaching test (shown in Fig. 10) of the cement samples showed a (+ or −) trend of pH values that correlate with their cement hydration efficiency shown in the compressive strength test results and the ICP-OES elemental analyses results (Fig. 8 and Fig. 11). The pH data show that at the cement paste sample with 5% clinoptilolite-bearing additive showed the “highest” pH value, whereas the cement sample with 15% mordenite-bearing additive showed the “lowest” pH value in the standardized leaching test. Though these small pH modifications are indications of the cementitious hydration characteristics efficiency of the solid waste samples (Rostamiparsa et al. 2023).

**Table 6:**
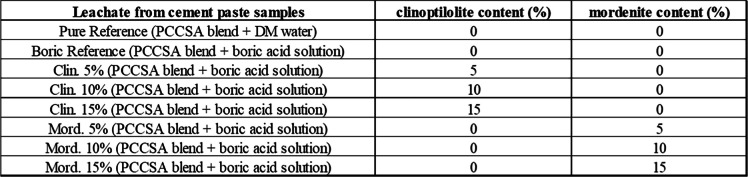
Sample descriptions of leachate collected during the standardized leaching test used for the pH, measure. Note: PCCSAC: 80% Portland cement + 20% sulfoaluminate cement; DM water: demineralized water; and boric acid solution: the simulated boric acid liquid waste solution (i.e., containing 40,000 ppm B + 400 ppm Cs + 400 ppm Ba)

### Elemental cesium and barium leached from the cementitious samples

The ICP-OES results of the leachates (Table 6) from cement paste samples obtained during the standardized leaching experiment (Fig. 11) measured the cumulative fraction concentration of elemental cesium and barium leached to assess the performance of each cement paste sample. The results show that the cement paste sample (Mord 15%) with 15% mordenite additive showed the highest cesium leachability (Fig. 11a, b), whereas the cement paste sample with 5% clinoptilolite additive shows the lowest cesium leachability (with only the reference sample showing a lower cesium leachability) (Fig. 11a, b).

**Fig. 12:**
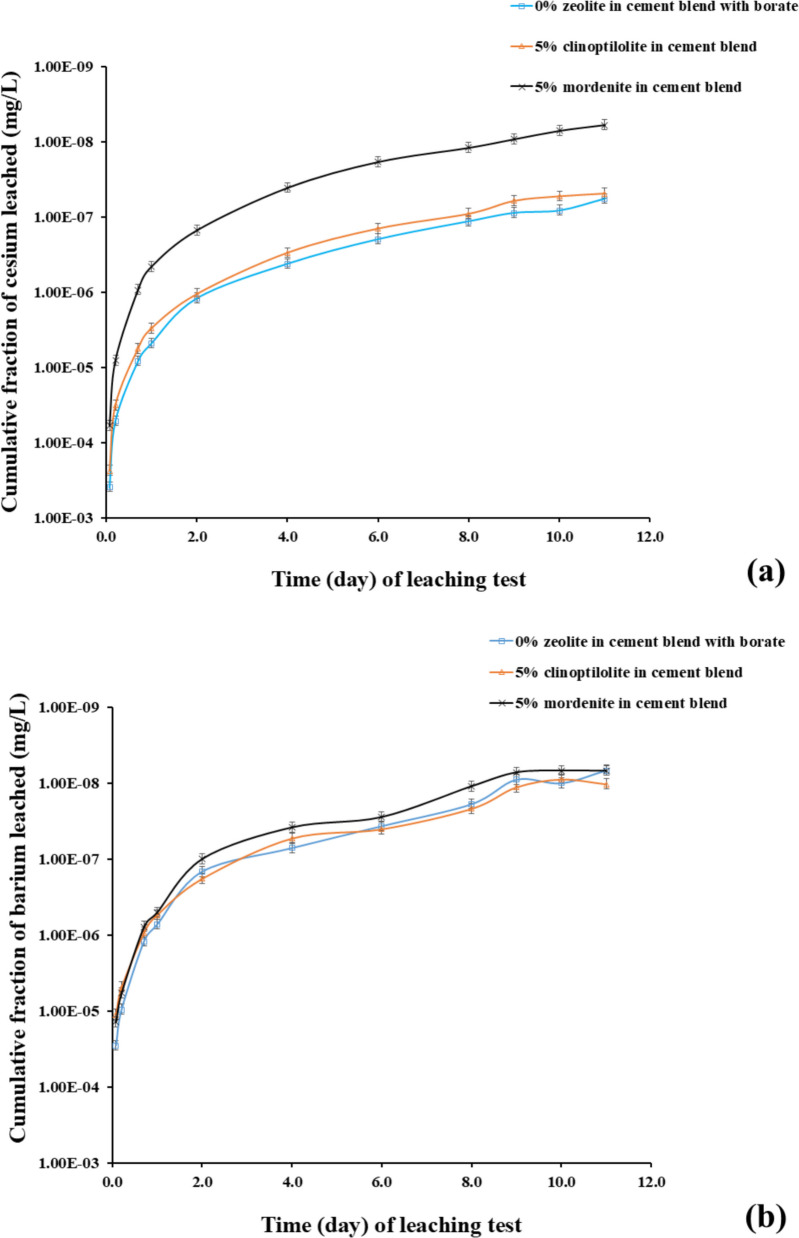
ICP-MS results of (**a**) cesium leachability from PCCSAC blend sample, 5% clinoptilolite cement-paste sample, and 5% mordenite cement-paste sample; against standardized leaching test time (**b**) barium leachability from PCCSAC blend sample, 5% clinoptilolite cement-paste sample, and 5% mordenite cement-paste sample; in function of leaching experiment time. The ICP-MS measurements for barium and cesium exhibited an average uncertainty of 6%

The ICP-MS results of the leachate samples (Table 6) collected during the leaching experiment (illustrated in Fig. 12) were utilized to quantify and further validate the CFL of elemental barium and cesium leached from the cement samples. The results compared barium and cesium leachabilities of the reference PCCSAC blend immobilizing borate (i.e., 0% zeolite) sample, 5% clinoptilolite cement-paste sample, and 5% mordenite cement-paste sample.

## Discussion

### Adsorption of Cs^+^and Ba^2+^in boric acid solution by the natural zeolite samples

**Fig. 13:**
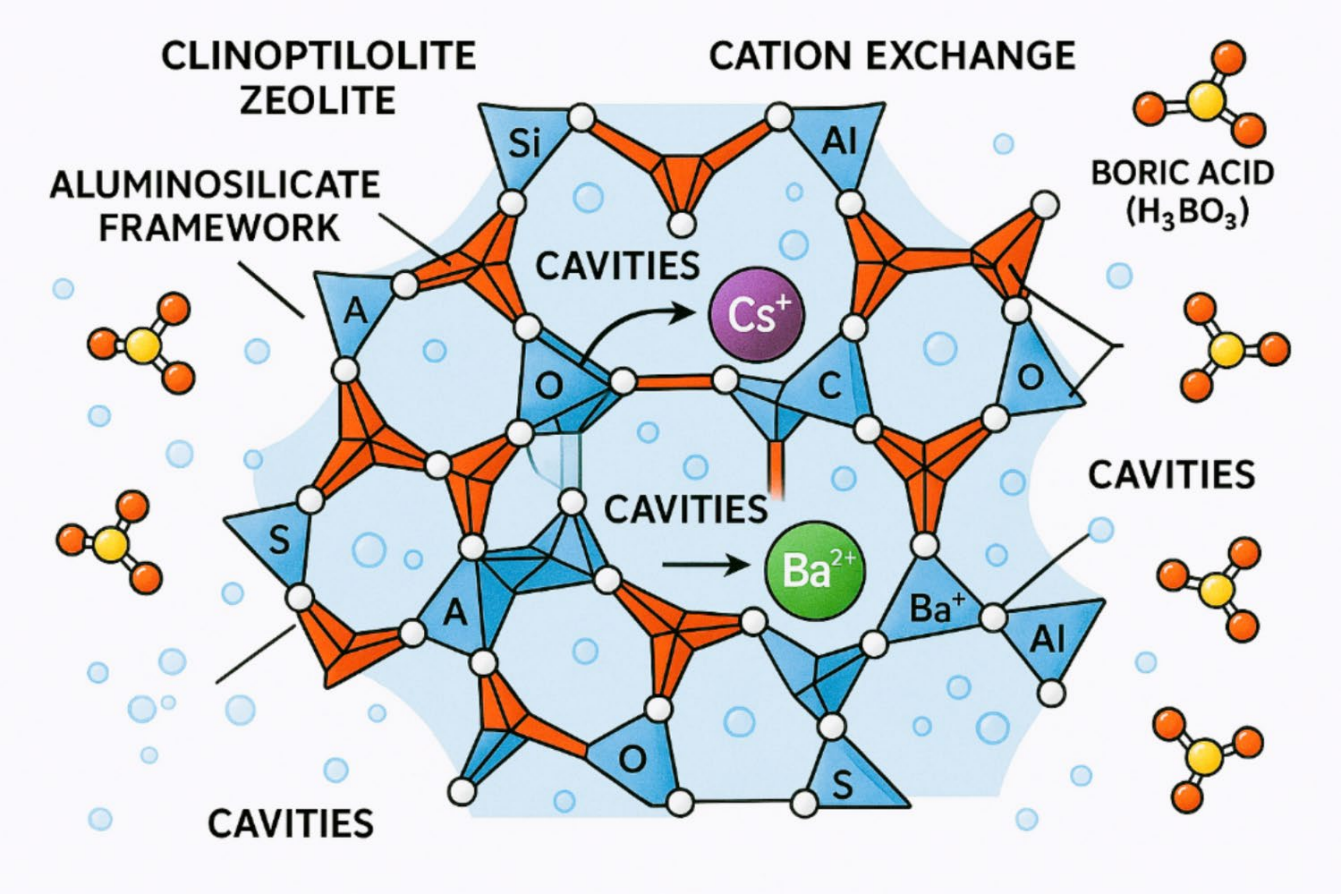
A diagrammatic representation of the cross-sectional view displaying the interior pore architecture and channel system of a clinoptilolite crystal structure undergoing cation exchange with cesium and barium ions in a boric acid solution. Blue tetrahedrals are representing SiO₄; red tetrahedra denote AlO₄; white/grey spheres signify oxygen atoms; purple spheres indicate Cs⁺; green spheres represent Ba^2^⁺; yellow elements correspond to the H₃BO₃ molecule; and directional arrows illustrate the ion exchange pathway

The comparative assessment batch experiment to study Cs^+^ and Ba^2+^ adsorption by clinoptilolite-bearing and mordenite-bearing samples, separately in the simulated boric acid-based waste solutions (Table 2 indicated that the untreated clinoptilolite-bearing samples showed the highest Cs^+^ adsorption rate in the boric acid – CsNO_3_ solution with 50.28 mg/L adsorption concentration and 25.83 mg/L adsorption rate in the boric acid – CsNO_3_ – Ba(NO_3_)_2_ solution (Table 2a). Conversely, the untreated mordenite-bearing samples showed comparatively lower values of Cs^+^ adsorption concentrations in both the boric acid – CsNO_3_ solution and boric acid – CsNO_3_ – Ba(NO_3_)_2_ solution, showing 5.80 mg/L and 6.66 mg/L, respectively (Table 2a). In terms of Ba^2+^ adsorption by the untreated zeolite-bearing samples, mordenite-bearing samples showed a higher adsorption capacity of 69.62 mg/L (Table 2c) in the boric acid – Ba(NO_3_)_2_ solution, whereas the clinoptilolite adsorbed 57.63 mg/L (Table 2c). However, in the boric acid – CsNO_3_ – Ba(NO_3_)_2_ solution clinoptilolite-bearing sample adsorbed 47.85 mg/L Ba^2+^ from the boric acid liquid waste solution; in contrast, the mordenite-bearing sample adsorbed 13.96 mg/L (Table 2c). The result demonstrates that both clinoptilolite-bearing and mordenite-bearing samples show the capacity to exchange ions with Cs^+^ and Ba^2+^ from the CsNO_3_ and Ba(NO_3_)_2_ solutions in the simulated boric acid waste solution, also releasing their additional major native cations (Na^+^, Ca^2+^, and K^+^) into the solution (Fig. 13). Therefore, it can be stated that the boric acid solution mainly facilitates a background electrolyte environment, which may influence the ion exchange process rather than participating in direct chemical reactions, whereas the clinoptilolite-bearing and mordenite-bearing adsorbates exchange their native cations with Cs^+^ and Ba^2+^ from their respective nitrate solutions in the main reactions (Abdel Maksoud et al. 2023; Kaminski et al. 2009; Kivan et al., 2024b; Sadeghi et al. 2021).

Both potassium hexacyanoferrate (II) – loaded (KCuHCF) clinoptilolite-bearing and mordenite-bearing samples showed a shift of both Cs^+^ and Ba^2+^ adsorption characteristics in the simulated waste solutions (Table 2). In the boric acid – cesium nitrate solution, Cs^+^ adsorption measurement showed that the clinoptilolite sample adsorbed 8.27 mg/L while the mordenite sample adsorbed 4.98 mg/L (Table 2b), whereas in the boric acid – cesium nitrate – barium nitrate solution, 21.0 mg/L Cs^+^ was adsorbed by the mordenite sample, while the clinoptilolite adsorbed 8.59 mg/L (Table 2b). In terms of Ba^2+^ adsorption by the KCuHCF-treated zeolites, the mordenite sample adsorbed 88.94 mg/L from the boric acid – cesium nitrate – barium nitrate solution, while the clinoptilolite adsorbed 85.18 mg/L (Table 2d) showing an increase of Ba^2+^ adsorption when compared with the untreated zeolite samples. Results of the KCuHCF-loaded zeolite samples indicated that through ion exchange, the potassium copper hexacyanoferrate (II) component adsorbed Cs^+^ selectively, releasing K^+^ into the boric acid solution. Ba^2+^ may be adsorbed by the KCuHCF-loaded zeolite samples via ion exchange, although this is unlikely to be as effective or selective for Cs^+^ (Cabaud et al. 2019; Loos-Neskovic et al. 2004).

Therefore, the result indicates that untreated clinoptilolite showed the greater Cs^+^ adsorption capacity in the borate waste solution in comparison with mordenite. However, loading the zeolites with KCuHCF did not improve the Cs^+^ adsorption from the borate solution but improved Ba^2+^ adsorption in the borate waste solution (indicated in Figs. 11 and 12).

### Physical characteristics of the cement waste forms

#### Surface morphology effects of the zeolite additives and boric acid liquid waste treatment

According to microstructural analysis of earlier cementitious matrix studies using SEM (Iklaga et al. 2025; Taylor 1990), efficiently reacted cement mineral phases appear darker in hue in SEM-BSE images; conversely, cement mineral phases like hatrurite, larnite, and brownmillerite, when not effectively reacted with the mixing solvent, appear brighter in hue. The result of this study (Fig. 6 and Fig. 7) indicates that the cement-paste samples with both zeolite additives (i.e., clinoptilolite and mordenite) showed more unreacted surface clinker minerals than the reference samples, except the cement-paste sample with 5% clinoptilolite, which indicated lesser unreacted surface clinker minerals, which serves as a semi-quantitative indicator of improved cement hydration. However, the cement-paste samples with mordenite additives proportionally showed the highest areas of unreacted surface clinker minerals, which indicates cement hydration retardation.

#### Effect of zeolite additives and boric acid liquid waste treatment on mechanical strength

The compressive strength experiment (Fig. 8) demonstrated that cement samples containing 5% untreated clinoptilolite exhibited higher mechanical strength measurements compared to all other cement samples in the research. When compared with compressive strength test results from our previous study (Iklaga et al. 2025), the cement-paste samples with zeolite additives generally showed lower compressive strength values. However, the cement-paste samples with 5% untreated clinoptilolite showed the lowest porosity effect (among samples with zeolite additives) as it indicated a value of approximately 25 N/mm^2^ when compared with the equivalent sample from the previous study which showed a 28 N/mm^2^ mechanical strength value (see Fig. 8). This significant decrease in compressive strength of the cement-paste samples with zeolite additives (except for 5% untreated clinoptilolite) when compared with the accepted standard and results from our previous study can be attributed to the microporous crystal structure and alumina oversupply by the zeolites’ additives. This leads to increased porosity of the cementitious matrices, cement hydration retardation, and overproduction of cement mineral phases like gypsum and ettringite which are deleterious to the mechanical structure of the cement-paste samples (Iklaga et al. 2025; Pimraksa and Chindaprasirt 2018). The cement-paste samples with potassium copper cyanoferrate (II) KCuHCF treated zeolites showed lower compressive strength values than the samples with untreated zeolite additives. The compressive strength measurement was compared against the European PC paste standard mechanical strength at 28-day range of 32.5–53 N/mm^2^ (European Committee for Standardization, 2016).

#### Effects of zeolite additives on the cement-paste samples’ mineralogy

The XRD comparative analyses of the cement-paste samples with clinoptilolite and mordenite additives (Fig. 9) indicated that the original cement mineral phases (i.e., alite, belite, and alumino-ferrite) in the clinoptilolite-added samples showed better cementitious hydration than in the mordenite-added samples when both were compared with the reference samples (i.e., cement-paste blend made with DM water and boric acid solution without zeolite additives, shown in Table 5). Ettringite production (produced by the hydration reaction of alite, gypsum, and ferrite) is optimized at a 5% clinoptilolite additive value, as belite hydration (from alumina supply, i.e., ye’elimite and tetracalcium aluminoferrite) is optimized. Ettringite is necessary for the regulation of early-age characteristics and cement setting. Both performance and durability are impacted by its stability and transformation (Taylor 1990). However, a greater than 5% clinoptilolite additive value leads to overproduction of ettringite, which could be deleterious (Kashaija et al. 2024) to the cementitious matrices along with gypsum observed in all the cement paste samples. In comparison with the mordenite-added cement-paste samples, the clinoptilolite-added samples show the characteristics of stable ettringite as it does not transform to monosulfoaluminate. This might be due to the excess sulfate supply from both the ye’elimite in the CSAC and gypsum supplied from the clinoptilolite-rich rhyolitic sample used as an additive. Gypsum overproduction in the 15% mordenite sample serves as a mineralogical indicator that it is the most chemically and physically unstable sample among all the cement wasteforms.

### Chemical characteristics of the leachates

#### Effects of pH on the cement-zeolite blend

The interaction of cement chemistry, zeolite reactivity, and boric acid speciation produces the pH-dependent complex hydrolysis of Portland cement (PC)-sulfoaluminate cement (CSAC) blends with clinoptilolite/mordenite additives in the presence of boric acid solutions (illustrated in Fig. 10). As the cement blend used for this study has a dominant PC (i.e., 80%) to CSAC (i.e., 20%) ratio, the high pH (i.e., 11.0 and greater) promotes the dissolution of silicate and aluminate cementitious mineral phases (i.e. alite and belite), producing portlandite (Glasser and Zhang 2001; Iklaga et al. 2025; Taylor 1990). Borates (B(OH)₄^⁻^) are increasing the porosity and delay the cementitious hydration by adsorbing to clinker mineral surfaces (Iklaga et al. 2025; Rostamiparsa et al. 2023). Borates precipitate as calcium borate phases (CaB(OH)₄^⁺^) at high pH values, which can immobilize contaminants such as Cs⁺ and Ba^2^⁺ in the cement matrices; however, this procedure can lead to the reduction of the cementitious wasteforms’ mechanical strength.

Additionally, at high pH in the PC dominated system, the clinoptilolite additive can dissolve and promote the pozzolanic reactions of SiO₂/Al₂O₃. However, because of contention between the Ca^2^⁺/Na⁺ in the alkaline pore solutions and Cs⁺/Ba^2^⁺, there is a reduction in the ion exchange efficiency of Cs⁺ uptake. Nevertheless, calcium borate can compensate by acting as a secondary immobilizing phase (El-Kamash et al. 2006). With CSAC addition in the blend (at pH ~ 10.0–11.5), as BaSO₄ precipitates and ettringite is produced, there is further immobilization. Thus, mordenite efficiently immobilizes Ba^2^⁺. Consequently, though zeolites refine pores by acting as micro-fillers, borate-induced retardation increases pore volume (porosity); and calcium borate may reduce leaching, but risk dissolving over time when exposed to carbonate or sulphate (B. Li et al. 2019). Hence, pH is an important factor that is an indicator of the physical and mineralogical variation (Figs. 6, 7, 8, and 9) observed between the clinoptilolite and mordenite added cement-paste wasteforms. Therefore, based on the preceding observations, the 5% clinoptilolite cement-paste indicates the optimal pH for cementitious Cs⁺ immobilization, while Ba^2^⁺ immobilization would be optimized in the ~ 10.0–11.5 pH range in the 5 (i.e., 7.5%) mordenite added cement-paste wasteforms.

#### Effects of zeolite percentage on cesium and barium leachability

Chemical complexations that impact the leachability of cesium (Cs⁺) and barium (Ba^2^⁺) are observed when clinoptilolite or mordenite are added to Portland cement (PC)-sulfoaluminate cement (CSAC) blends in the presence of borate solutions (as indicated in the ICP-OES and ICP-MS results illustrated in Figs. 11 and 12 respectively). Borate-induced calcium borate precipitates may immobilize Cs⁺, partially balancing the leachability, as borate anions can reduce clinoptilolite’s Cs⁺ adsorption capacity by ~ 25% because of competition for exchange sites (Dyer 2007). The larger molecular pores (crystal structure) in mordenite influence the preservation of Ba^2+^ adsorption in the presence of borate anions, while borate–sulfate competition prevents BaSO₄ (barite) precipitation in the CSAC component of the cementitious matrices. Stable borate complexes, such as BaB(OH)₄^⁺^, can be formed by Ba^2^⁺, which decreases mobility with increasing alkalinity (El-Kamash et al. 2006; B. Li et al. 2019). The result indicated that the 5% clinoptilolite cement-paste sample showed negligible cesium leachability difference with the reference sample, while the 5% mordenite cement-paste sample indicated increasing cesium leachability. Both zeolite-added cement-paste samples indicated negligible barium leachability when compared with the reference sample.

## Conclusions

To achieve optimal immobilization of Cs and Ba in boric acid liquid waste, it is necessary to balance the factors influencing the cementitious waste forms, the zeolite additive type/content, the borate concentration, and the cement mix ratios. This is required to minimize Cs and Ba leachability while the cementitious wasteforms maintain physical and chemical integrity. The factors of borate concentration and cement mix ratios had been dealt with in our previous study (Iklaga et al. 2025). For this study, the physical and chemical characteristics of the zeolite-bearing additives and the cement-paste samples were investigated, and the following conclusion can be inferred:

- The untreated clinoptilolite sample showed the most significant Cs^+^ adsorption capacity in the borate solution in comparison with the mordenite sample. Loading the clinoptilolite and mordenite with KCuHCF did not improve the Cs^+^ adsorption from the borate waste solution. On the other hand, the KCuHCF treatment improved the Ba^2+^ adsorption in the borate waste solution as barium is a more electropositive cation.
- The cement-paste samples with untreated 5% clinoptilolite-bearing additive indicated optimal physical characteristics when compared to both the reference cement-paste sample and cement-paste samples with mordenite-bearing additive. The 15% mordenite-added cement-paste samples showed the worst physical characteristics.
- The XRD analysis of the cement samples has shown that an addition of 5% clinoptilolite in the PC/CSAC blend exhibited enhanced mineralogical stability for borate immobilization. The 15% mordenite-added cement-paste samples showed the worst chemical characteristics.
- Chemical assessment via Cs^+^ and Ba^2+^ leachability analyses showed that the cement-paste sample with 5% clinoptilolite-bearing additive showed negligible Cs leachability compared to the reference sample, while the cement-paste sample with 5% mordenite-bearing additive indicated increased Cs leachability. Both zeolite-added cement-paste samples indicated negligible barium leachability when compared with the reference sample.

Hence, the overall results in this study indicate that the cement-paste wasteforms with 5% untreated clinoptilolite-bearing additive are both physically and chemically optimal for the immobilization of Cs and Ba in boric acid liquid waste when compared to the cement-paste samples with mordenite-bearing additive. Based on the results of this study, it is recommended that future experiments study clinoptilolite additive ratios within closer decimal ranges to the optimal ratio.

## Supplementary Information

Below is the link to the electronic supplementary material.

ESM 1 Supplementary Material 1 (DOCX 29.5 KB)

## Data Availability

All the data is published in the paper.
