# Supplementary material for: Optimized cementitious immobilization of simulated cesium and barium radionuclides in borate waste solution by natural zeolite additives
Source: Environ Sci Pollut Res Int. 2026 Jan 29;33(6):2388–408. doi: 10.1007/s11356-025-37369-1 (PMC12960504; doi:10.1007/s11356-025-37369-1)
Supplement: Supplementary file 1 — Supplementary Material 1 (DOCX 29.5 KB) [file 11356_2025_37369_MOESM1_ESM.docx]

**Supplementary information**

**Supplementary Table 1:** Portland cement (PC) and calcium sulfoaluminate cement (CSAC) paste chemical and mineralogical specifications supplied by CEMKUT Ltd (Iklaga et al., 2025). (LOI: loss on ignition; IR: insoluble residue)

| **Chemical compositions of major and minor elements in PC and CSAC clinkers** | | | | | | | | | | | | | | | | | |
| --- | --- | --- | --- | --- | --- | --- | --- | --- | --- | --- | --- | --- | --- | --- | --- | --- | --- |
| **Oxides** | SiO2 | Al2O3 | Fe2O3 | CaO | | MgO | K2O | | Na2O | | SO3 | Cl^-^ | TiO2 | | IR | LOI | |
| Mass percentage  (m/m %)  in **PC** | 19.21 | 5.83 | 3.62 | 65.75 | | 2.40 | 0.26 | | 0.02 | | 3.43 | 0.02 | 0 | | 0 | 1.26 | |
| Mass percentage  (m/m %)  in CSAC | 6.18 | 23.49 | 1.28 | 36.64 | | 4.80 | 0.36 | | 0.75 | | 21.54 | 0 | 0.36 | | 0.94 | 0.70 | |
| **Solid phase composition** | | | | | | | | | | | | | | | | | |
| **Mineralogical**  **name (technical formula)** | | **alite**  **(C3S)** | | | **belite (C2S)** | | | **aluminate (C3A)** | | **ye'elimite (C4A3S̅ )** | | | | **ferrite (C4AF)** | | | **Anhydrite**  **(CS̅)** |
| Chemical formula | | Ca3SiO5 | | | Ca2SiO4 | | | Ca3Al2O6 | | Ca4(AlO2)6SO4 | | | | Ca4Al2Fe2O10 | | | CaSO4 |
| Mass percentage  (m/m %) in **PC** | | 57.5 | | | 11.8 | | | 9.3 | | 0 | | | | 11.0 | | | 5.8 |
| Mass percentage  (m/m %) in **CSAC** | | 1.7 | | | 44.6 | | | 0 | | 16.1 | | | | 13.2 | | | 24.4 |
| **Uncertainty (repeatability and reproducibility) (Gabry, this table is related to the first one!! Why did you mixt them up?)** | | | | | | | | | | | | | | | | | |
| **Component** | | **Test standard** | | | | | | **Repeatability (%)** | | | | | | **Reproducibility (%)** | | | |
| **LOI** | | EN 196-2:2013 | | | | | | 0.04 | | | | | | 0.08 | | | |
| **SO_3_** | | EN 196-2:2013 | | | | | | 0.07 | | | | | | 0.08 | | | |
| **IR** | | EN 196-2:2013 | | | | | | 0.04 | | | | | | 0.06 | | | |
| **SiO_2_** | | EN 196-2:2013 | | | | | | 0.10 | | | | | | 0.25 | | | |
| **Fe_2_O_3_** | | EN 196-2:2013 | | | | | | 0.08 | | | | | | 0.15 | | | |
| **Al_2_O_3_** | | EN 196-2:2013 | | | | | | 0.10 | | | | | | 0.25 | | | |
| **CaO** | | EN 196-2:2013 | | | | | | 0.15 | | | | | | 0.43 | | | |
| **MgO** | | EN 196-2:2013 | | | | | | 0.21 | | | | | | 0.25 | | | |
| **Cl^-^** | | EN 196-2:2013 | | | | | | 0.005 | | | | | | 0.01 | | | |
| **Na_2_O** | | EN 196-2:2013 | | | | | | 0.01 | | | | | | 0.02 | | | |
| **K_2_O** | | EN 196-2:2013 | | | | | | 0.02 | | | | | | 0.03 | | | |

**Supplementary Table 2:** Preparation specifications of cement waste forms for cementitious immobilization of boric acid liquid waste with the following natural zeolite additives: **ZCS** = clinoptilolite-bearing sample; **ZMS** = mordenite-bearing sample; **PCCSAC** = cement blend + DM water; + boric acid; **5** = cement blend + 5% zeolite additive; **10** = cement blend + 10% zeolite additive; **15** = cement blend + 15% zeolite additive.

| **Cement Sample ID** | **PC share (%)** | **CSAC share (%)** | **ZC share (%)** | **ZM share (%)** | **Boron conc. (mg/l)** | **Cesium conc. (mg/l)** | **Barium conc. (mg/l)** |
| --- | --- | --- | --- | --- | --- | --- | --- |
| **PCCSAC** | 80 | 20 | 0 | 0 | 0 | 0 | 0 |
| **ZFS** | 80 | 20 | 0 | 0 | 40000 | 300 | 300 |
| **ZCS5** | 76 | 19 | 5 | 0 | 40000 | 300 | 300 |
| **ZCS10** | 72 | 18 | 10 | 0 | 40000 | 300 | 300 |
| **ZCS15** | 68 | 17 | 15 | 0 | 40000 | 300 | 300 |
| **ZMS5** | 76 | 19 | 0 | 5 | 40000 | 300 | 300 |
| **ZMS10** | 72 | 18 | 0 | 10 | 40000 | 300 | 300 |
| **ZMS15** | 68 | 17 | 0 | 15 | 40000 | 300 | 300 |

**Supplementary Table 3:** Different zeolites or zeolite-bearing rocks and boric acid liquid waste as adsorbents were used for the batch adsorption experiment. Abbreviations: ZC = pure clinoptilolite-bearing zeolite; ZM = pure mordenite-bearing zeolite; ref = reference; DM = demineralized water; T = zeolite-bearing samples treated with HCl (hydrochloric acid); L = zeolite-bearing samples treated with HCl and KCuHCF (potassium copper hexacyanoferrate (II) trihydrate + copper (II) sulphate).

| **Sample**  **Names** | **Zeolite type** | **Mixing solvent** | **B concentration**  **(mg/l)** | **Cs concentration**  **(mg/l)** | **Ba concentration**  **(mg/l)** | **HCl treated**  **(T)** | **KCuHCF loaded**  **(L)** |
| --- | --- | --- | --- | --- | --- | --- | --- |
| ZCrefT | clinoptilolite | DM | 0 | 0 | 0 | Yes | No |
| ZCBaT | clinoptilolite | DM | 40000 | 0 | 300 | Yes | No |
| ZCCsT | clinoptilolite | DM | 40000 | 300 | 0 | Yes | No |
| ZCBaCsT | clinoptilolite | DM | 40000 | 300 | 300 | Yes | No |
| ZCrefL | clinoptilolite | DM | 0 | 0 | 0 | Yes | Yes |
| ZCBaL | clinoptilolite | DM | 40000 | 0 | 300 | Yes | Yes |
| ZCCsL | clinoptilolite | DM | 40000 | 300 | 0 | Yes | Yes |
| ZCBaCsL | clinoptilolite | DM | 40000 | 300 | 300 | Yes | Yes |
| ZMrefT | mordenite | DM | 0 | 0 | 0 | Yes | No |
| ZMBaT | mordenite | DM | 40000 | 0 | 300 | Yes | No |
| ZMCsT | mordenite | DM | 40000 | 300 | 0 | Yes | No |
| ZMBaCsT | mordenite | DM | 40000 | 300 | 300 | Yes | No |
| ZMrefL | mordenite | DM | 0 | 0 | 0 | Yes | Yes |
| ZMBaL | mordenite | DM | 40000 | 0 | 300 | Yes | Yes |
| ZMCsL | mordenite | DM | 40000 | 300 | 0 | Yes | Yes |
| ZMBaCsL | mordenite | DM | 40000 | 300 | 300 | Yes | Yes |
